# Coupling Computational Homogenization with Crystal Plasticity Modelling for Predicting the Warm Deformation Behaviour of AA2060-T8 Al-Li Alloy

**DOI:** 10.3390/ma16114069

**Published:** 2023-05-30

**Authors:** Ali Abd El-Aty, Sangyul Ha, Yong Xu, Yong Hou, Shi-Hong Zhang, Bandar Alzahrani, Alamry Ali, Mohamed M. Z. Ahmed

**Affiliations:** 1 Department of Mechanical Engineering, College of Engineering at Al Kharj, Prince Sattam Bin Abdulaziz University, Al Kharj 11942, Saudi Arabia; 2 Mechanical Engineering Department, Faculty of Engineering-Helwan, Helwan University, Cairo 11795, Egypt; 3 PKG Simulation, SK Hynix Inc., Icheon 17336, Gyeonggi, Republic of Korea; dubuking@postech.ac.kr; 4 Shi-Changxu Innovation Center for Advanced Materials, Institute of Metal Research, Chinese Academy of Sciences, Shenyang 110016, China; shzhang@imr.ac.cn; 5 School of Materials Science and Engineering, University of Science and Technology of China, Shenyang 110016, China; 6 Department of Materials Science and Engineering & RIAM, Seoul National University, Seoul 08826, Republic of Korea; yonghou@snu.ac.kr

**Keywords:** AA2060-T8, crystal plasticity modelling, computational homogenization, dynamic recovery, storage and recovery processes

## Abstract

This study aimed to propose a new approach for predicting the warm deformation behaviour of AA2060-T8 sheets by coupling computational homogenization (CH) with crystal plasticity (CP) modeling. Firstly, to reveal the warm deformation behaviour of the AA2060-T8 sheet, isothermal warm tensile testing was accomplished using a Gleeble-3800 thermomechanical simulator at the temperatures and strain rates that varied from 373 to 573 K and 0.001 to 0.1 s^−1^. Then, a novel crystal plasticity model was proposed for describing the grains’ behaviour and reflecting the crystals’ actual deformation mechanism under warm forming conditions. Afterward, to clarify the in-grain deformation and link the mechanical behaviour of AA2060-T8 with its microstructural state, RVE elements were created to represent the microstructure of AA2060-T8, where several finite elements discretized every grain. A remarkable accordance was observed between the predicted results and their experimental counterparts for all testing conditions. This signifies that coupling CH with CP modelling can successfully determine the warm deformation behaviour of AA2060-T8 (polycrystalline metals) under different working conditions.

## 1. Introduction

One of the most critical lightweight metallic materials used recently in aircraft, military, and aerospace industries is the third generation Al-Li alloys due to their remarkable mechanical and physical properties compared with conventional Al alloys. Adding Li caused these outstanding properties, impacting the modulus of elasticity and weight reduction. Adding 1% of Li reduced and enhanced the density and modulus of elasticity of Al alloy by 3% and 6%, respectively [1,2,3]. Al-Li alloys are categorized into three generations based on their manufacture date: first (1st), second (2nd), and third generations (3rd) [1,4]. AA2060-T8 is one of the third generation alloy families introduced in the last few years by Alcoa Inc. to replace the traditional Al alloys in aerospace applications [5]. Nevertheless, it shows poor formability and serious anisotropic behaviour notably at room temperature, limiting its industrial applications [6,7,8,9]. High strain rate deformation [10,11,12,13,14,15,16,17,18] and high temperature forming [19,20,21] are significant techniques for enhancing the formability of AA2060 sheets and addressing the drawbacks of conventional cold forming technologies. Thus, understanding the warm deformation behaviour of AA2060 experimentally and using advanced modeling approaches under different working conditions is essential for manufacturing sound components from AA2060-T8 alloy.

So far, little investigations have been accomplished to figure out the flow behaviour and deformation mechanisms of this alloy at high temperatures. Abd El-Aty et al. [22,23] investigated the mechanical behaviour of AA2060 at room temperature and a wide range of strain rates. Furthermore, they proposed a new computational framework to determine this alloy’s flow and anisotropic behaviours at room temperature and several strain rates. After that, they [24] developed an Arrhenius constitutive model to figure out the effect of strain rates and temperatures on the flow behaviour of AA2060 and estimated its activation energy (Q) to reveal the difficulties of forming this alloy under warm forming conditions. Ou et al. [25] explained the hot deformation behaviour of AA2060 at different forming conditions, and they realized that dynamic recovery (DRV) is the main reason for softening during hot forming. Additionally, they mentioned that 380–500 °C and 0.01–3 s^−1^ are the optimum hot forming conditions (temperatures and strain rates) for AA2060-T8 alloy sheets. After that, Gao et al. [26] combined their outstanding results [25] to examine the capability of fabricating aerospace components from AA2060 by hot forming in-die quenching (HFQ) technology. They mentioned that 2 s^−1^ and 470 °C are the optimum HFQ conditions (strain rate and temperature) to manufacture sound AA2060-T8 components.

From the above discussion, it is clear that figuring out the flow behaviour of AA2060 at warm forming conditions experimentally and theoretically was not yet studied. The flow behaviours of Al alloys under warm forming conditions are complex since they depend on many factors (i.e., strain rates, strain, and deformation modes) [21]. These key factors control the dynamic softening and strain hardening, affecting the formability and deformation behaviour of these alloys [27,28]. Dynamic softening mechanisms, such as DRV, usually appear in Al and Al-Li alloys with high stacking-fault (SFE) during warm and hot deformation [28]. Elkhodary et al. [29], Pandey et al. [30], Abedrabbo et al. [31], Clayton et al. [32], and Lin et al. [21] reported that the mechanisms that contribute to softening and recovery during deformation at high temperatures are susceptible to strain rate and temperature. Thus, predicting the warm deformation behaviours of AA2060-T8 sheets is meaningful for describing their mechanical responses at several working conditions. Therefore, the motivation of this study was to couple computational homogenization (CH) and crystal plasticity (CP) modelling to propose a new framework that considers the storage and recovery processes for simulating and predicting the warm deformation behaviours of AA2060 at various deformation conditions.

Three essential features are required to simulate and predict the macroscopic mechanical behaviour by coupling CH and CP modeling. The first feature is the constitutive equation that characterizes the grains’ behaviour for reflecting the crystal deformation mechanisms. The second feature represents the actual microstructure AA2060-T8 sheets using RVE. The third feature is the material parameters that are crucial for characterizing the behaviours of grains correctly. This investigation used CP modeling as the constitutive model for AA2060 sheet grains. In the proposed CP model, the total dislocation density was decomposed into edge and screw components to consider DRV caused by a cross slip of the screw dislocations at low homologous temperatures. The experimental stress-strain curves characterized the material parameter of the proposed CP model. After that, the proposed constitutive model was implemented using a monolithic time-integration algorithm-based backward Euler method. For considering the in-grain deformation behaviours, the homogenization process used the representative volume element (RVE) for representing the real sheet metal microstructure. For constructing RVE, the initial microstructures and micro-textures were measured by electron backscatter diffraction (EBSD). In addition, periodic boundary conditions (PBC) were developed for considering geometrical and deformation-induced anisotropy. The accuracy of the proposed approach (coupling CH with CP modeling) for predicting the warm deformation behaviour of AA2060 at different conditions was verified by comparing the experimental and predicted results. Additional verification was performed by calculating a set of statistical parameters for quantitatively evaluating the reliability and measuring the predictability of the proposed approach.

## 2. Experimental Procedure

**Figure 1:**
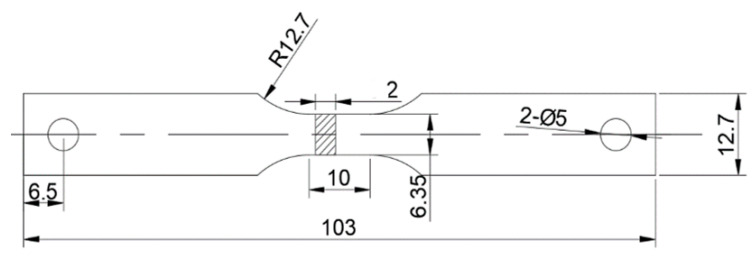
The details of the tensile sample of AA2060T8 sheet (2 mm thickness) used for the Gleeble simulator.

The material utilized in the current study was rolled sheets of AA2060-T8. A Gleeble-3800 simulator was utilized to accomplish the isothermal warm tensile tests at 373, 423, 473, 523, as well as 573 K and $\dot{\varepsilon }=0.001,\;0.01,\;\mathrm{and}\;0.1$ s^−1^ using the sample depicted in Figure 1.

**Figure 2:**
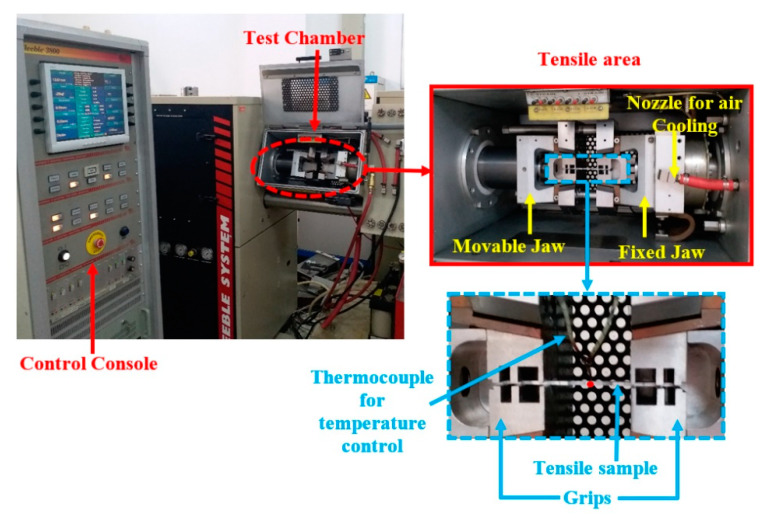
The description of the tensile testing zone of the Gleeble-3800 material simulator.

**Figure 3:**
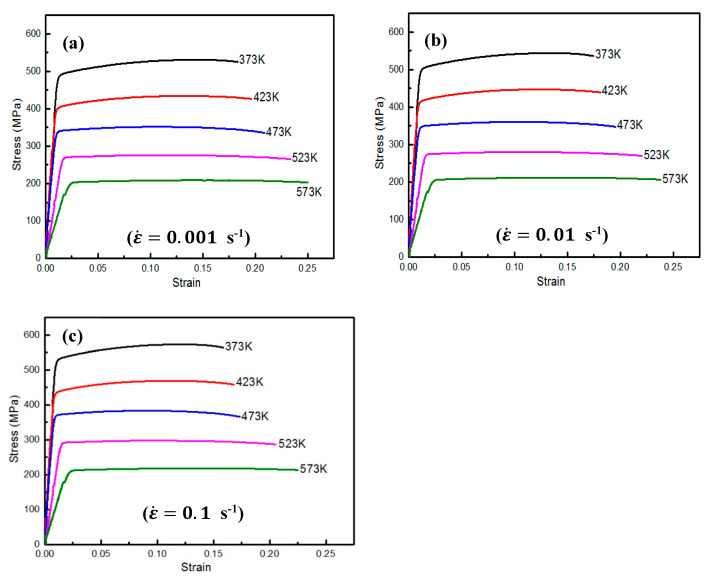
The (σ−ε) curves of AA2060 at testing temperatures of 373 K, 423 K, 473 K, 523 K, and 573 K and $\dot{\varepsilon }$ (**a**) 0.001, (**b**) 0.01, and (**c**) 0.1 s^−1^.

The Gleeble-3800 simulator was designed to perform the physical simulations of thermomechanical processes at a maximum heating rate, quenching rate, and stroke rate of 10,000 °Cs^−1^, 10,000 °Cs^−1^, and 2000 mms^−1^, respectively. Furthermore, the Gleeble-3800 simulator is outfitted with a control system for imposing the aggressive decay of the speed of the actuator to achieve a constant strain rate. The setup of the Gleeble-3800 simulator used in this study is shown in Figure 2. The deformation temperature was evaluated using thermo-couples that were welded in the gauge length center of the test sample. This provided a signal for precise checking, as presented in Figure 2. The deformation temperatures, strain, and strain rates were automatically controlled and noted. All the test samples were heated to the necessary testing temperatures before testing. To ensure repeatability and consistency, all test samples were repeated three times at each test condition, and the average value was considered for each condition. The experimental stress-strain (σ-ε) curves of AA2060-T8 sheets obtained from the isothermal warm tensile tests using Gleeble material simulators under the aforementioned warm forming conditions are depicted in Figure 3.

## 3. Coupling CH with CP Modelling and Numerical Implementation

Several items are necessary to predict the warm deformation behaviour of AA2060 via coupling CH with CP modelling. These items are a new constitutive equation describing the grain’s behaviour for reflecting the deformation mechanism of crystals under warm working conditions, RVE for representing grains of the AA2060-T sheets, and a set of parameters for precisely describing the grain’s behaviour. The details of each feature are discussed in the following sub-sections.

### 3.1. CP Modeling

The present study used a dislocation-based single CP model as a constitutive equation of AA2060. The theoretical framework of the proposed CP model followed the previous works performed by Cheong et al. [33], Arsenlis et al. [34], and Ha et al. [35]. The kinematic framework of finitely deforming the single crystal was introduced based on multiplicative decomposition. After that, a description of stress states and the evolution of three kinetic variables were presented (i.e., crystallographic slip shearing rates, slip resistance, and dislocation densities). The multiplicative decomposition described the kinematics of an elasto-plastically deforming continuum body by introducing a locally stress-free intermediate configuration $\tilde{\beta }$ that is not uniquely defined within a rigid body rotation. However, we constitutively prescribed that the plastic flow of the crystalline materials left the crystal lattice undistorted and un-rotated. Thus, the deformation gradient F, as written in Equation (1), can be decomposed into an elastic $F^{e}$ part and a plastic $F^{p}$ part [36], where Fe contains rigid body rotation and the elastic distortion of the lattice, and $F^{p}$ describes the plastic flow associated with the crystallographic slip.

(1) $$F=F^{e}F^{p}$$

Due to the incompressibility condition during the plastic deformation, the following conditions are required.

(2) $$J^{p}=det\;F^{p}=1$$

By similarity, ***L*** (velocity gradient tensor) is also decomposed to $\left(L^{e}\right)$ and ($L^{p})$, elastic and plastic elements, respectively, as follows:

(3) $$L=L^{e}+L^{p}$$

where $L^{e}$ and $L^{p}$ are the elastic and plastic velocity gradients written as:

(4) $$L^{p}=F^{e\;}{\tilde{L}}^{p}F^{e-1\;}$$

${\tilde{L}}^{p}$ is the pull-back of the plastic velocity gradient to the intermediate configuration $\;\tilde{\beta }$.

The velocity gradient tensor **L** is described as follows:

(5) $$L={\dot{F}}^{e}F^{e-1}+F^{e\;}{\tilde{L}}^{p}F^{e-1\;}\;$$

At low homologous temperatures, it is assumed that the dislocation glide on well-defined crystallographic slip systems is the dominant source of plastic deformation. Thus, the plastic flow can be written as:

(6) $${\tilde{L\;}}^{p}={\dot{F}}^{p}F^{p-1}=\sum_{\alpha -1}^{n}{\dot{\gamma }}^{a}{𝕊}^{a}$$

where:

(7) $${𝕊}^{a}=m_{0}^{a}⊗n_{0}^{a}\;$$

where ${\dot{\gamma }}^{a}$ stands for the shearing rate on the *α*-slip system, and *n* is the potential slip system’s number. ${𝕊}^{a}$ is the Schmid tensor that describes the slipping systems with the direction and slip-plane normal $m_{0}^{a},\;n_{0}^{a}$, respectively [36,37,38]. For FCC metallic materials, the crystallographic slip was expected to take place on the {111} 〈110〉 slipping systems. The elastic deformation totally carries the distortion of the crystallographic slip systems. Thus, the slipping direction ($m^{\alpha }$) in the current configuration is characterized to its form in $\tilde{\beta }$ by:

(8) $$m^{\alpha }=F^{e}m_{0}^{\alpha }$$

In addition, $n^{\alpha }$ (i.e., the equivalent slip plane normal) was written as:

(9) $$n^{\alpha }=n_{0}^{\alpha }F^{e-1}$$

where it is assumed that $m_{0}^{\alpha }$ and $n_{0}^{\alpha }$ are orthogonal to each other.

Since the elastic deformations are infinitesimal compared with the plastic deformation, the linear Saint Venant relation can be used to describe the stress response of single crystals:

(10) $$S^{e}=C^{e}:E^{e}$$

where $S^{e}$ is the second Piola-Kirchhoff stress tensor and is related to the Cauchy stress tensor (σ) as:

(11) $$S^{e}=J^{e}F^{e-1}\sigma F^{e-T}$$

and ***E*** is the elastic Green-Lagrange strain tensor defined as:

(12) $$E^{e}=\left(\;C^{e}-I\right)/2$$

where:

(13) $$C^{e}=F^{eT}\;F^{e}$$

$C^{e}$ can be fully characterized by temperature-dependent stiffness parameters [39]:

(14) $$C_{ij}=C_{ij}^{o}-\frac{S_{ij}}{\exp\left(\frac{t_{ij}}{\theta }\right)-1}$$

where θ is the absolute temperature (K). $C_{11}^{0}=114.2\;GPa,\;C_{12}^{0}=61.9\;GPa,\;C_{44}^{0}=31.6\;GPa$, $S_{44}=2.56\;GPa$, $S_{12}=2.0\;GPa,$ and $S_{11}=10.1\;GPa,\;t_{11}=258.4;\;t_{12}=293.6,\;t_{44}=168$. $S_{11}$,$\;S_{12}$, and $S_{44}$ are the elastic compliance parameters defined by

(15) $$S_{11}=\frac{C_{11}+C_{12}}{\left(C_{11}-C_{12}\right)\left(C_{11}+2C_{12}\right)}$$

(16) $$S_{12}=\frac{-C_{12}}{\left(C_{11}-C_{12}\right)\left(C_{11}+2C_{12}\right)}$$

(17) $$S_{44}=\frac{1}{C_{44}}$$

In the intermediated configuration, the resolved shear stress tensor $\left(\tau^{\alpha }\right)$ projected on the slip plane in the glide direction can be written as:

(18) $$\tau^{\alpha }=(C^{e}S^{e}):{𝕊}^{a}$$

where $C^{e}$ is the elastic right Cauchy-Green deformation tensor.

The Orowan equation can be used to relate the crystallographic slip shearing rate with the collective motions of dislocations, as follows:

(19) $${\dot{\gamma }}^{\alpha }=b\rho^{\alpha }{\bar{v}}^{\alpha }$$

where b is the magnitude of the Burgers vector, and $\rho^{\alpha }$ and ${\bar{v}}^{\alpha }$ are the density and average velocity of mobile dislocations on the α slip system.

Under a quasi-static condition, the average dislocation velocity can be approximated as:

(20) $$\bar{v}≅l_{f}/t_{w}$$

$l_{f}$ is the average distance between each obstacle on the slip plane, and $t_{w}$ is the waiting time for a dislocation to surpass the local obstacles. When a dislocation segment conflicts with an obstacle, the frequency of the jumps over the obstacles through a thermal fluctuation at a finite temperature θ>0 is given by the following:

(21) $$t_{w}{}^{-1}=v_{D}\;b\;l_{f}\left[exp\left(-\frac{\Delta G^{*}}{k_{B}\theta }\right)\right]$$

where $v_{D}$ is the Debye frequency, $k_{B}$ is the Boltzmann constant, and $\Delta G^{*}$ is the difference in the activation-free enthalpy when the dislocation segment moves from the stable configuration to the unstable configuration. Kocks et al. [38] proposed the following form:

(22) $$\Delta G^{+}=F_{0}{\left\{1-{\langle \frac{\left|\tau^{\alpha }\right|-s_{a}^{\alpha }}{{\hat{\tau }}^{\alpha }}\rangle }^{\;p}\right\}}^{q}$$

For detecting the shapes of the profile of the energy barrier associated with the interaction between the obstacle and dislocation [39], ***p*** and ***q*** are considered to be within the following range:

(23) 0≤p≤1, and 1≤q≤2

By combining Equations (19)–(22), the evolution of the crystallographic shearing rates is expressed as:

(24) $${\dot{\gamma }}^{a}={\dot{\gamma }}_{0}\;exp\left[\frac{F_{0}}{k_{B}\theta }{\left\{1-{\langle \frac{\left|\tau^{\alpha }\right|-S_{a}^{\alpha }}{{\hat{\tau }}^{\alpha }}\rangle }^{p}\right\}}^{q}\right]\;$$

where ${\dot{\gamma }}_{0}$ is the reference slip rate.

The evolutions of the total dislocation density are written as the competition of the storage and recovery mechanisms:

(25) $$\dot{\rho }={\dot{\rho }}^{\left(+\right)}+{\dot{\rho }}^{\left(-\right)}\;$$

Assuming the dominant source of the dislocation nucleation, the athermal storage rate can be written as:

(26) $${\dot{\rho \;}}^{\alpha }=\frac{c{\dot{\gamma }}^{\alpha }\;}{bL^{\alpha }}\;$$

where c denotes the fraction of the shearing rate by the total dislocation, and L is the mean free path.

(27) $${\dot{\rho \;}}^{\alpha }=2y_{c}\frac{\rho^{\alpha }{\dot{\gamma }}^{\alpha }\;}{b}$$

where $y_{c}$ is the critical annihilation distance for canceling out the two dislocations with opposite polarities.

For the annihilation process, the cross slip of screw dislocations and the climb of edge dislocations are responsible for the dynamic recovery at low and high temperatures, respectively. According to Essman et al. [39], the screw dislocations glide, multiplicate, and cross in the interior of dislocation cells, whereas edge dislocations accumulate in the cell walls.

Thus, we further decompose the total dislocation density as:

(28) $$\rho^{\alpha }=\underset{edge\;dislocation\;compoents}{\underbrace{\rho_{e}^{\alpha }}}+\underset{screw\;dislocation\;components}{\underbrace{\rho_{s}^{\alpha }}}\;$$

where $\rho_{e}^{\alpha }$ and $\rho_{s}^{\alpha }$ are edge and screw dislocation density components, respectively.

Therefore, the evolution of dislocation densities can be expressed as:

(29) $${\dot{\rho }}_{i}^{\alpha }=\frac{C_{i}}{b}\left[\frac{K_{i}}{L_{i}}-2d_{i}\rho_{i}^{\alpha }\right]\left|{\dot{\gamma }}^{\alpha }\right|\;,\;i=e,s$$

Since we are concerned with the deformation response at low homologous temperatures, the critical annihilation distance for edge dislocations is assumed to be constant for simplicity. Since cross slip is a thermally activated process, however, the critical annihilation distance for screw dislocations is also a function of temperature and stacking fault energy. Motivated by the form proposed by Nix et al. [40], we employed the following phenomenological form:

(30) $$y_{s}={\hat{y}}_{s}exp\left(-\frac{b}{a\theta }\right)$$

where ${\hat{y}}_{s}$ is the reference critical annihilation distance (${\hat{y}}_{s}=400\;nm)$, and a and b are constants, a = 0.001, b = 8.617 × 10^−5^.

According to the Taylor equation [41,42], the total athermal slip resistance parameters $\left(S_{T\;}^{\alpha }\right)$ were calculated via:

(31) $$S_{a\;}^{\alpha }=\lambda \mu b\sqrt{\sum_{\beta =1}^{N_{s}}h^{\alpha \beta }\rho^{\beta }}\;$$

where μ is the shear modulus, ***b*** is the Burgers vector’s magnitude, and *λ* is the statistics parameter to identify the deviations from the regular spatial arrangements of the dislocation densities. Additionally, $h^{\alpha \beta }$ is the matrix of dislocation interaction. Many scholars introduced various models depending on the discrete dislocation dynamics [43,44,45] or the back extrapolation of latent hardening experimentation [46,47]. Nevertheless, in this investigation, the equation used was Equation (32) for simplicity:

(32) $$h^{\alpha \beta }=\omega_{1}+\left(1-\omega_{2}\right)\delta^{\alpha \beta }\;$$

where $\delta^{\alpha \beta }$ is the Kronecker delta, and $\omega_{1}$ and $\omega_{2}$ are the interaction coefficients.

### 3.2. CH Procedures

**Figure 4:**
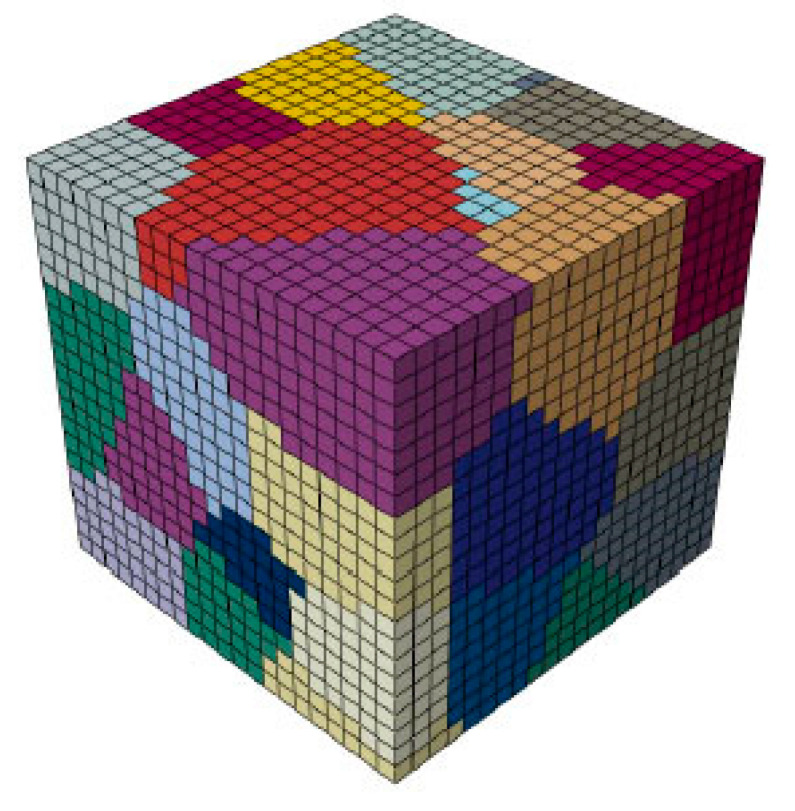
The proposed RVE model (grain-based) utilized in the current investigation, where the cubic elements depicted the grain.

CH is primarily based on the characteristics and the finite element (FE) modeling of the RVE, which represents the actual microstructure of AA2060. In the CH of metallic material, three models of RVEs were used. In the current study, a grain-based RVE model was utilized, in which every grain is represented by several cubic elements. Thus, further information about the size and the shapes of the grains in the metallic materials can be efficiently included. In this investigation, the established RVE model possessed equiaxed grains shapes. Furthermore, the model was discretized 100 × 100 × 100 C3D8R element. Figure 4 depicts the RVE model utilized in the present investigation. This model consists of fifty grains and various colors representing the different grains. A neper 4.6.0 software [48] used widely to establish a polycrystal model was used in the current study to create the unit cell model. According to the statistics, as the grain numbers of RVE models increased, their responses converged to the actual macroscopic behaviour of the material.

**Figure 5:**
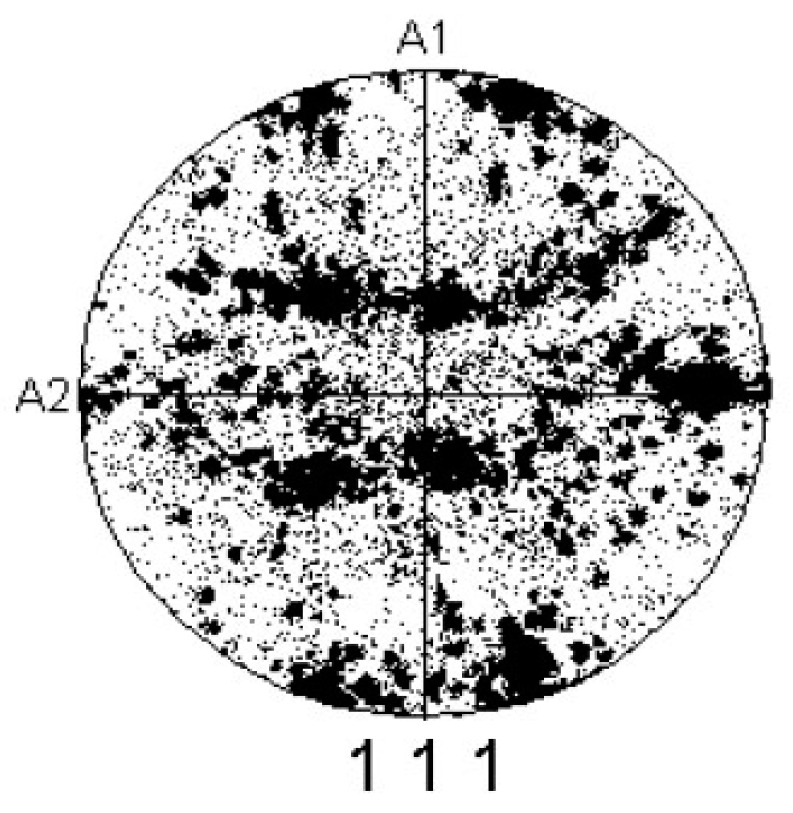
The (111) pole figure describing the initial texture of AA2060-T8 for fifty grains.

The main issue when establishing the grain-based RVE models is representing the initial textures of the received material. Most previous studies, for simplicity, assumed that all the materials had isotropic behaviours [49,50,51]. Nevertheless, due to thermomechanical processing, most as-received sheet metals exhibit anisotropic behaviour, where the deformation histories are basically unknown. Hence, in the current investigation for correctly assigning the initial textures for the RVE models, the crystallographic data collected through EBSD measurements were reduced using the coarsening method, eliminating the pixel every two pixels and reducing the point numbers in a data set by four. This method was recurred for acquiring fifty crystallographic orientations approximating the initial textures of AA2060. The size of the grains, besides the texture distributions, might affect the macroscopic behaviour of the RVE models; however, the surface measurements detected using EBSD cannot precisely reflect the sub-surface distributions of grains for actual material. Notwithstanding, as presented in the consequent results, the macroscopic *(*σ−ε*)* figures might be reasonably obtained when the element numbers in the RVE model are acceptable. Figure 5 depicts the (111) pole figure of the reduced texture of the initial AA2060-T8.

The mechanical behaviour of the RVE model was obtained by FE modelling, where the established model was used directly as FE meshes, in which each voxel describes one cubic finite element. Additionally, appropriate boundary conditions must be implemented on each face of the cubic element. Hazanov et al. [49] noticed in their study that the deformation behaviours of the metallic sheets determined via implementing the PBCs on the cubic cell’s faces were very close to the actual behaviour than that obtained from imposed displacements or forces. Hence, in the current investigation, PBCs were used for considering the geometrical and deformation-induced-anisotropy caused by the initial texture of AA2060. Additionally, PBCs were utilized to ensure compatibility between adjacent boundaries of the RVE model before and after deformation. It should be noted that, in the case of anisotropic metallic materials, even if the macroscopic external loading is uniform, the deformation of the RVE may be non-uniform.

To accurately predict the deformation behaviour of the AA2060 sheet utilized in this study under warm forming conditions by coupling CP modelling with CH framework, it was crucial to determine a set of material parameters that are necessary to properly describe the behaviour of grains and are utilized in the proposed dislocation density-based CP model. The set of parameters included $C_{s\;}$, $\;C_{e}$, $K_{e\;}$, $\;K_{s\;}$, $d_{e}$, and $d_{s}.\;C_{s}$ and $C_{e}$ represent the fractions of the total slip rate contributions by screw and edge segments, respectively. $K_{e}$ and $K_{s}$ are constants that govern the mobility of dislocations, while $d_{e}\;$ and $d_{s}$ denote the maximum distances for mutual annihilation between the antiparallel edge and screw dislocations, respectively. Previous studies determined these parameters based on calibration results for Al alloys. From these parameters, only $K_{s\;}$ and $d_{s}$ were adjustable for approximating the stress-strain behaviour. The set of material parameters for pure Al single crystals was adopted as a baseline, and it was assumed that precipitation due to Li particles could account for the hardening rate of AA2060. However, the slip resistance of different slip systems in the grains of polycrystalline aggregates can be dissimilar due to varying hardening during previous deformation. For simplicity, it was assumed that dislocations were evenly distributed, and the initial slip resistance of each slip system was the same. Additionally, the initial slip resistance was set to be higher than that of pure Al single crystals due to precipitation hardening.

## 4. Verification of the Proposed Approach

**Figure 6:**
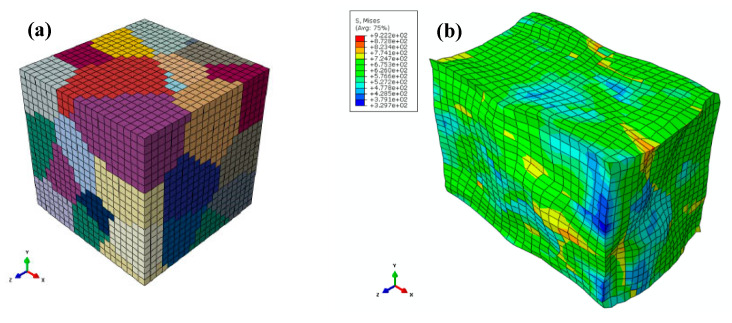
The (**a**) undeformed and (**b**) deformed RVE model.

**Figure 7:**
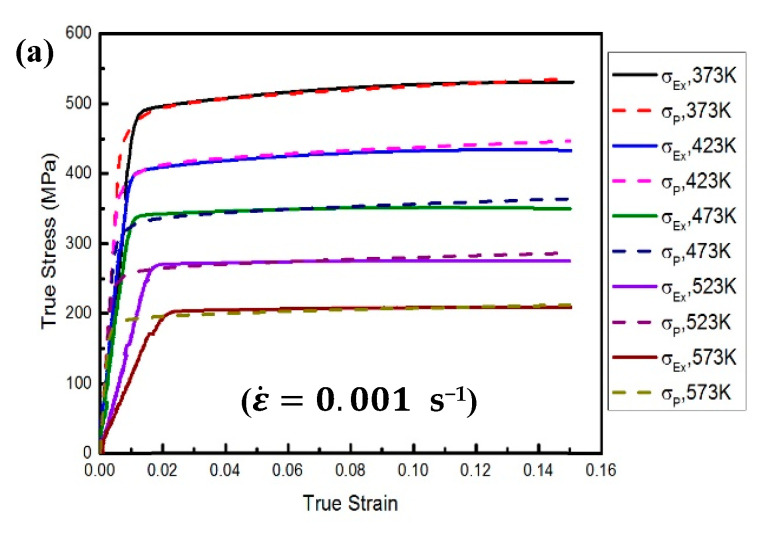

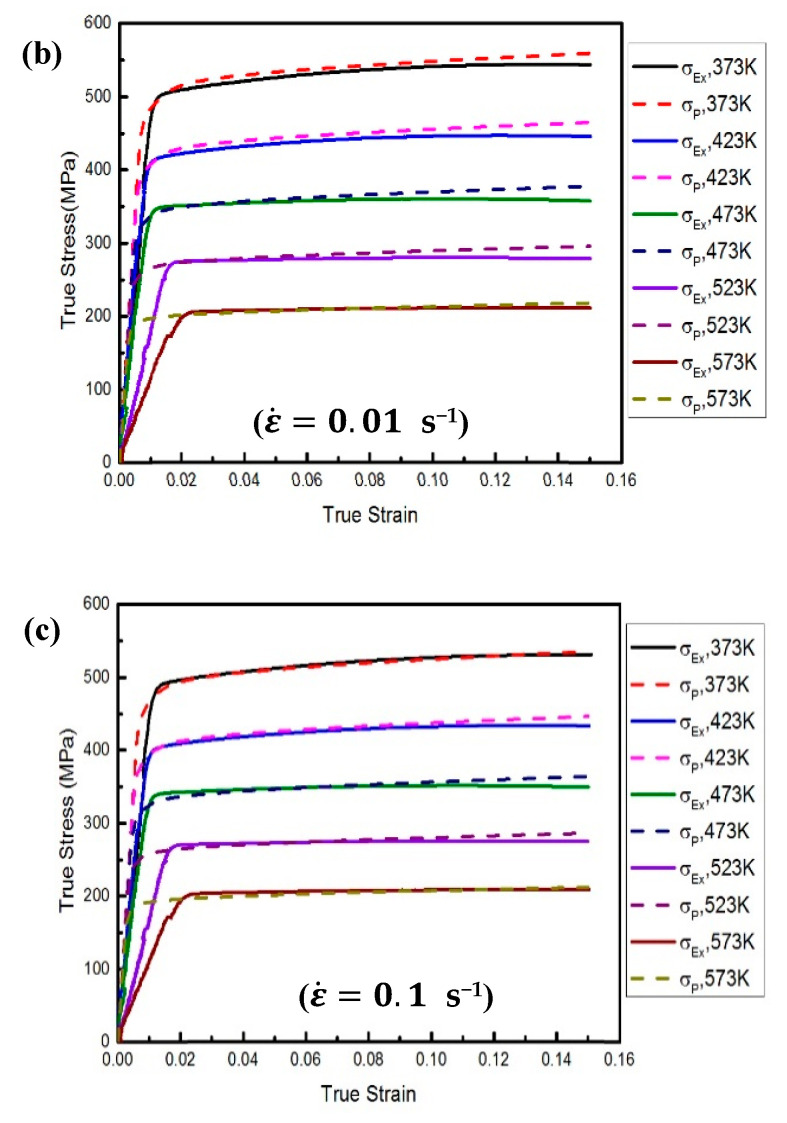
The correlation between the predicted (σ−ε) curves and their counterpart collected from tensile testing at $\dot{\varepsilon }$ of (**a**) 0.001, (**b**) 0.01, and (**c**) 0.1 s^−1^.

The deformation behaviour of AA2060 sheets under warm forming conditions was predicted and captured by the FE modeling of the proposed RVE model. The grain’s behaviour for reflecting the actual deformation mechanisms was modelled via CP modelling. Figure 6 demonstrates the RVE model before and after 15% uniaxial deformation. Figure 6b shows the heterogeneous deformation that happened in each grain due to the interaction impacts between the adjacent grains. The accuracy and the predictability of coupling CH with CP modelling for predicting the warm deformation behaviour of the AA2060-T8 sheet were validated by comparing the stress-strain curves determined from the proposed approach with those acquired from experimental work, as depicted in Figure 7. As presented in these figures, the predicted results aligned with those obtained from experimentation in all experimental conditions. This signifies that coupling CH with CP modelling, which considers the phenomenon of dynamic recovery, can predict the warm deformation behaviour of AA2060-T8 sheets. This remarkable agreement was attributed to including the physical mechanisms of the plastic deformation and the key details of AA2060-T8′s microstructure in the proposed approach.

**Figure 8:**
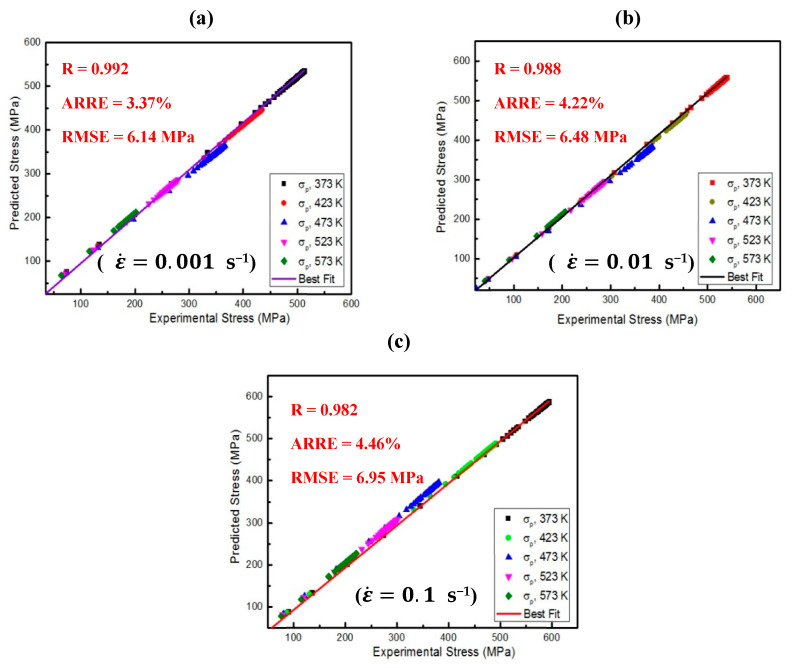
The comparison between determined stresses and their counterparts obtained from experimentation at various deformation conditions (i.e., 373, 423, 473, 523, and 573 K) and $\dot{\varepsilon }$ of (**a**) 0.001, (**b**) 0.01, and (**c**) 0.1 s^−1^.

Further validation for the predictability of coupling CH with CP modeling was performed by calculating statistical parameters such as ***NMBE****, **RMSE***, ***AARE***, and ***R***, as listed in Table 1 and introduced in Figure 8. ***R*** is the correlation coefficient, which is a vital parameter calculated from Equation (33) to determine the strength of linear relations between predicted and experimental stresses. If the ***R*** nears 1, this signifies that the reliability of the proposed approach is outstanding [50,51].

(33) $$R=\frac{\sum_{i=1}^{i=N}\left(\sigma_{E}^{i}-{\bar{\sigma }}_{E}\right)\left(\sigma_{P}^{i}-{\bar{\sigma }}_{P}\right)}{\sqrt{\sum_{i=1}^{i=N}{\left(\sigma_{E}^{i}-{\bar{\sigma }}_{E}\right)}^{2}\sum_{i=1}^{i=N}{\left(\sigma_{P}^{i}-{\bar{\sigma }}_{P}\right)}^{2}}}$$

***N*** is the number of points utilized in this analysis. $\sigma_{E}^{i}$ and $\sigma_{P}^{i}$ are the experimental and predicted stresses, respectively. ${\bar{\sigma }}_{E}$ and ${\bar{\sigma }}_{P}$ are the mean of the experimental and predicted stresses, respectively. Equations (34) and (35) were used to determine the average absolute relative error (***AARE****)* and root mean square error (***RMSE****),* which are important for quantifying the capability of the proposed approach for predicting the warm deformation behaviour of the AA2060-T8 sheet correctly [52,53,54]. The small values of ***AARE*** imply that coupling CH with CP modeling can determine that the warm reliability of the proposed approach is significant and vice versa [55].

(34) $$AARE\;\left(\%\right)=\frac{1}{N}\;\sum_{i=1}^{i=N}\left|\frac{\sigma_{E\;}^{i}-\sigma_{P}^{i}}{\sigma_{E.\;}^{i}}\right|\times 100$$

(35) $$RMSE=\sqrt{\frac{1}{N}\sum_{i=1}^{i=N}{\left(\sigma_{E}^{i}-\sigma_{P}^{i}\right)}^{2}}$$

The last statistical parameter used in this validation stage was normalized mean bias error (***NMBE****),* utilized to quantify the mean bias in predicted results. ***NMBE*** was calculated from Equation (36), where the positive value of ***NMBE*** means over-prediction; however, the negative value indicates under-prediction [55].

(36) $$NMBE\;\left(\%\right)=\frac{(1/N)\sum_{i=1}^{i=N}\left(\sigma_{E}^{i}-\sigma_{P}^{i}\right)}{(1/N)\sum_{i=1}^{i=N}\sigma_{E.}^{i}}\times 100$$

## 5. Conclusions

In the current study, A new approach was proposed by coupling CH with CP modelling for predicting the warm deformation behaviour of AA2060 and $\dot{\varepsilon }$ of (a) 0.001, (b) 0.01, and (c) 0.1 s^−1^. For revealing the warm deformation behaviour of the AA2060 sheet, isothermal tensile testing was accomplished using a Gleeble-3800 thermomechanical simulator at temperatures and strain rates that varied from 373 to 573 K and 0.001 to 0.1 s^−1^. Then, a novel CP model was proposed for describing the grains’ behaviour and reflecting the crystals’ actual deformation mechanism under warm forming conditions. In the CP model, the total dislocation density was decomposed into edge and screw components to consider DRV caused by a cross slip of the screw dislocations at low homologous temperatures. The experimental stress-strain curves characterized the material parameters of the proposed CP model. After that, the proposed constitutive model was implemented using a monolithic time-integration algorithm-based backward Euler method. Afterward, to clarify the in-grain deformation and link the mechanical behaviour of AA2060-T8 with its microstructural state, RVE elements were created to represent the microstructure of AA2060-T8, where several finite elements discretized every grain. A remarkable accordance was observed between the predicted results and their experimental counterparts for all testing conditions. This signifies that coupling CH with CP modelling can determine the warm deformation behaviour of AA2060-T8 (polycrystalline metals) under different working conditions.

## Figures and Tables

**Table 1:** R, ARRE%, NMBE%, and RMSE calculated at $\dot{\varepsilon }\;$(0.001, 0.01, and 0.1 s^−1^).

Value	R	ARRE (%)	RMSE (MPa)	NMBE (%)
$\dot{\varepsilon }=0.001$/s	0.992	3.37	6.14	0.186
$\dot{\varepsilon }=0.01$/s	0.988	4.22	6.48	0.192
$\dot{\varepsilon }=0.1$/s	0.982	4.46	6.95	0.199

## Data Availability

Data are available upon request through the corresponding author.
